# Response to Letter to the Editor

**DOI:** 10.1097/JS9.0000000000003509

**Published:** 2025-09-10

**Authors:** Mohammad Taheri, Aria Baniahmad

**Affiliations:** a Institute of Human Genetics, Jena University Hospital, Jena, Germany; b Research Institute for Urology and Nephrology, Shahid Beheshti University of Medical Sciences, Tehran, Iran

We sincerely thank Han *et al*^[1]^. for their thoughtful comments on our recent study regarding the “The lncRNAs PART1 and ADAMTS9-AS2 act in an antithetic manner on AR signaling and induction of cellular senescence in prostate cancer cells”^[2]^. Their remarks underscore important directions for future research.

Our analyses indicate that during prostate cancer progression, *PART1* expression is elevated in tumor tissues and downregulated by supraphysiological androgen levels (SAL) used in the bipolar androgen therapy, being considered as a tumor suppressive mechanism. Functionally, by SAL treatment *PART1* dampens AR transcriptional output, consistent with its proposed role as an AR co-repressor, thereby restraining AR-driven senescence programs. This dual effect suggests that *PART1* may act as a molecular “brake,” shifting the balance between proliferation and senescence. Importantly, sustained *PART1* expression may confer a survival advantage by allowing tumor cells to evade AR-induced senescence, highlighting its potential as a therapeutic target.

Regarding combinatorial therapy, modulation of *PART1* – together with AR pathway inhibitors (ARPIs) – may enhance treatment efficacy. In particular, suppressing *PART1* could sensitize tumors to AR antagonists by preventing its inhibitory effect on AR-driven senescence. Conversely, therapeutic upregulation of *PART1* might be relevant in contexts where excessive AR signaling drives tumor proliferation, emphasizing the context-dependent role of this lncRNA.

We also agree that targeting the ADAMTS9-AS2/miR-7-5p/CAV1 axis holds promise for synergizing with bipolar androgen therapy. Ongoing mechanistic studies in our laboratory aim to clarify whether manipulating the interplay between *PART1* and *ADAMTS9-AS2* can optimize senescence induction and help overcome drug resistance in castration-resistant prostate cancer.

In summary, we greatly appreciate the constructive feedback, which reinforces the translational potential of our findings and motivates further studies to define the therapeutic utility of lncRNA directed interventions in prostate cancer.

The TITAN Guidelines 2025 – Governing Declaration and Use of AI have been completed and are included in the supplementary materials, as required^[3]^.

## Data Availability

Not applicable.
